# Physiological anthropology and human biology: overlapping interests, differing approaches

**DOI:** 10.1186/s40101-026-00441-x

**Published:** 2026-08-06

**Authors:** Douglas E. Crews

**Affiliations:** Department of Anthropology, School of Public Health, 4034 Smith Laboratory, Columbus, OH 43210-1106 USA

## Abstract

This brief review describes multiple overlapping research interests within the fields of human biology and physiological anthropology, for example, human physiological responsiveness to environmental stressors, e.g., heat, cold, hypoxia, constructed environments, and workloads, while also exploring how research approaches, goals, and methodologies often differ significantly across these related disciplines. To begin, this contribution reflects on how following publication of Damon’s 1975 edited volume *Physiological Anthropology*, research on physiological anthropology, expanded and flourished within North American biological anthropology and human biology, and within physiological anthropology, specifically Japan. Since Damon’s volume, human biologists and physiological anthropologists have explored multiple topics of mutual interest as reflected in recent publications within the Journals of Physiological Anthropology and the American Journal of Human Biology. By viewing and exploring modern human physiological diversity as responses to experienced physical, social, and ecological settings, including interactions with our constructed, engineered, manufactured, and technological environments, physiological anthropology addresses a broad research agenda. Here, I briefly review several areas of overlapping research, sleep, cold, and aging, within human biology and physiological anthropology, wherein specific goals, methods, and technologies differ.

## Introduction

Sharing academic roots within physical/biological anthropology, research directions within human biology and physiological anthropology have diverged over time. In addition to sharing academic and historical roots, human biology and physiological anthropology share research interests in human physiological variation, adaptability, and adaptedness to current, historical, and evolutionary environments and experienced sociocultural stressors. Although having multiple overlapping research interests, they often differ in research emphases, goals, and methods for exploring ranges of, causes for, and mechanisms underlying modern human variation. What continues to unite them is their overlapping research emphases on human adaptability and adaptive responses to experienced environments, impacts of sociocultural stressors on human biology, physiology, and variation, and the influences of expanding technology on health and survival.

To do so, I briefly review several overlapping and divergent research interests, analytic methods, and goals within physiological anthropology and human biology as illustrated by publications in recent volumes of their associated journals: *American Journal of Human Biology* (AJHB), *Journal Physiological Anthropology* (JPA), and the *American Journal of Biological Anthropology* (AJBA). This directed review is not intended to address all or even most of the extensive research published across these journals. Rather, it is intended to explore the expansion of physiological anthropology across the world as an independent branch of biological anthropology, a branch partly overlapping and coequal with human biology. It begins with a brief review of the development of physiological anthropology in the USA following Damon’s edited volume *Physiological Anthropology* [1] published in 1975. This is followed by a review of articles published in recent volumes of the AJBA, AJHB, and JPA illustrating their differing, but often overlapping, approaches to understanding modern human diversity, health, adaptability, and adaptation. Topics of continuing research interest to both physiological anthropologists and human biologists.

This brief contribution is not all inclusive. Rather, it is a descriptive look at current research directions within and across two related anthropological subdisciplines. It is designed to illustrate overlapping and differing research interests, methodologies, experimental designs, sampling strategies, research scopes, and goals. Both disciplines conduct field and laboratory research to determine how modern human physiological and physical variation, health, and well-being reflect human responsiveness to environments our ancestors inhabited and the natural, built, and sociocultural settings in which we reside today.

Many materials cited herein were retrieved from articles published in recent issues of the AJBA, AJHB, and JPA, along with related journals. These provide an overview of research activities across biological anthropology (BA), highlight commonalities, and allow us to explore differences in research designs, goals, sampling, and research methodologies, for example, emerging research in the field of human factors anthropology, techno-anthropology, exploring manufacturing, human factors engineering, built environments, human-techno interactions, anthropological ergonomics, particularly within physiological anthropology (PA), and human biology (HB), wherein the scope of research is broad and often overlapping.

Although research goals, methods, and sampling strategies often differ, both PA and HB examine adaptive and maladaptive human physiological responses to life’s stressors, e.g., altitude, climate, culture change, diet, natural light, temperature, and aging. Human biologists are more likely to conduct field research in populations experiencing specific stressors (e.g., extreme cold or heat, cultural and dietary changes, hypoxia, insecurity, malaria) using biomedical, laboratory, physical, and physiological assessments (e.g. allostatic load, blood pressure, body habitus, frailty, lipid profiles); participate in field research within differing populations and settings; and employ participant self-reported surveys to assess sociocultural, dietary, and environmental stressors. Physiological anthropologists also research these topics. However, their interests extend to behavioral, physiological, and genetic responses to manufactured and technological stimuli, e.g., artificial lighting, noise, machinery, engineered environments, manufactured and technological products, workloads, and techno-adaptability.

Although closely allied, human biology and physiological anthropology differ [2]. Foremost, human biology is a subfield within biological anthropology, while physiological anthropology in Japan is an independent academic field with an organized academic organization established approximately half a century ago, and publishing the *Journal of Physiological Anthropology (JPA)*. Similarly, although a subfield within North American Biological Anthropology, the Human Biology Association is an independent scientific organization publishing the *American Journal of Human Biology* (AJHB) for almost a century. Both human biologists and physiological anthropologists research distributions, functions, and benefits of human physiological variation across natural, constructed, sociocultural, and technological environments. Physiological anthropologists collaborate more extensively with industry in developing and testing products for human use, e.g., automobile interiors, cellphones, clothing, keyboards, and safety wear. As noted by Yasukouchi [2], research on human physiological response to environmental and manufactured stressors published in the *Journal of Physiological Anthropology* overlaps significantly with publications in the *American Journal of Human Biology*. Browsing recent issues of both, shared themes range across age-related physiological variation, anthropometry, body weight/mass, brown adipose tissue, circadian rhythms, growth and development, illness/disease, metabolism, physical activity, responsiveness to cold, heat, and hypoxia, energetics, genes, and sleep (see, for example, AJHB 36:8–11, 2024; JPA 43:1–4, 2024). Both disciplines view human variation as reflective of adaptative (genetic) responses to previous and current environmental stressors, along with individual and population adaptability (phenotypic responsiveness) to unique environmental and sociocultural settings. Both viewpoints are integral to understanding how responses to previous and current stressors differentially structure physiological variation among populations today. Contrary, to extensive overlap in research topics and analytic methods published in the AJHB and JPA, publications in neither overlap extensively with those in recent issues of the *American Journal of Biological Anthropology* (see, for example, AJBA 185:1–4, 2024). Therein, publications center archaeology, bioarchaeology, dentition, fossil hominins, genetics, ill-health in past populations, nonhuman primates, and paleontology. Few describe modern human physiological variation. A strikingly different observation than reported by Ice [3] 23 years ago, when 43% of publications in AJBA explored physiological variation among living humans.

As described by contributors to Damon’s 1975 edited volume *Physiological Anthropology* [1] and Baker’s description of Human Population Biology [4, 5], within the USA, research on human responsiveness to environmental and sociocultural stressors is an established transdisciplinary, laboratory, and field-based endeavor within human biology. Human population biologists continue to explore how such stressors influence modern human physiological adaptability and its underlying genetic basis. Physiological anthropologists often differ from human biologists in their research goals, study designs, and sampling strategies as they focus more on human-technology interfaces, “techno-adaptability,” and applied human science [6]. Both disciplines assess physiological changes, e.g., blood pressure and oxygenation, body temperature, cognitive responsiveness, heart rate, pupil dilation, and sleep, in response to environmental and sociocultural stressors. However, physiological anthropologists are more likely to explore these topics while conducting applied research on functional adaptability to engineered, technological, and manufactured products in controlled laboratory, industrial, and ergonomic settings. Human biologists generally conduct their research in naturally stressful environmental, socioecological, and cultural settings to determine differentials in human stressor responses.

Over their evolutionary history, humans have experienced and survived extensive environmental, socioecological, and sociocultural stressors. They have responded to these by evolving adaptable and malleable physical, physiological, and psychological responses (see [7]). Long before having developed modern technological responses to stressors, evolved adaptability supported human survival and reproductive success across the world’s extreme environments, from arctic cold to equatorial heat, and hypoxic altitudes. They explored new environments and experienced previously unknown stressors that altered their existing gene-environment balances, lifeways, physiology, and phenotypes [2, 6, 7]. Thus, human populations of the present vary significantly in their physiological and morphological adaptability and responsiveness to experienced environmental stressors, technologies, and lifeways than did their ancient ancestors.

## Physiological anthropology in the USA

In the late twentieth century, the edited volume *Physiological Anthropology* [1] sparked a growth of research in physiological anthropology across North America. Therein, thirteen topical chapters explored the breadth and expanding interests of physiological research within human biology. Following an introduction by Paul T. Baker, topical chapters included human responses to cold, exercise, heat, light, noise, and nutrition, along with human performance, workloads, and behavioral ecology. Areas of continuing research interest today across human biology, physiological anthropology, and physiology as are aging, circadian rhythms, nutrition, physical performance, sleep, and responsiveness to stress.

Noted for his contributions to human biology and physiological anthropology in North America and globally, Paul T. Baker (1927–2007) emphasized evolutionary and ecological approaches to understanding human variation while developing the transdisciplinary subfield of human population biology (HPB) within human biology (see [4, 8, 9]). HPB explores variation in physiological function and body morphology across human populations with the goal of identifying phenotypic acclimatization, adaptability, and genetic adaptations to experienced environments. Baker viewed modern individual and populational physiological variation as reflecting an eternal triangle of biological, environmental, and cultural interactions [4, 8–10]. Exploring such interactions, Baker documented morphological and physiological responses, and suggested possible evolved responses to stressors within and across populations, cultures, and environments.

### Human biology and physiological anthropology

Damon’s [1] vision of physiological anthropology as a worldwide research and applied endeavor is today’s reality. Within North American biological anthropology today, physiological anthropology is represented most clearly in the American Journal of Human Biology wherein 90%-plus of recent publications report at least one, and commonly multiple assessments of human physiological variation (see, for example, AJHB 36:8–11). As physiological anthropologists do, human biologists document population adaptations and physiological adaptability to environmental and sociocultural stressors, cold, heat, hypoxia, solar radiation, aging, artificial lighting, human performance, occupational stress, and workloads. Research by human biologists also is published frequently across biology, epidemiology, and physiology journals (e.g., [10–15]). Internationally, physiological anthropologists collaborate with manufacturing and industry in developing accessible and ergonomic product designs for human use, while also exploring influences of developing technologies on human physiological function, adaptability, and adaptive responses. A focus centered more on adaptability to environmental and technological stressors, polytypism, and functional potentiality, along with variable physiological capacities, mechanisms, and coordination (see [6]). Conversely, human biologists focus more on human physical, psychosocial, and physiological adaptability to experienced environmental and sociocultural stressors, e.g., aging, allostatic load, chronic and communicable disease, cold, heat, frailty, illness, morbidity, and mortality.

Human biologists frequently collaborate with nonanthropological specialists in ecology, genetics, medicine, and cultural anthropology to conduct transdisciplinary research designed to understand how environmental, ecological, and sociocultural settings pattern human physiology, health, and survival. This research ranges from populations experiencing extremes of cold, heat, hypoxia, solar radiation, and workloads, to changing social, cultural and ecological settings, and age-related physiological decline. To survive and reproduce, all mammals must regulate their body temperature, sleep to maintain health and reproduce, and survive increasing physiological and functional declines with increasing age. Here, recent research on human sleep, response to cold exposure, and aging are reviewed briefly to illustrate several overlapping and variable approaches to modern human variation within human biology and physiological anthropology.

#### Sleep

That all animals sleep supports the conclusion that sleep is necessary for maintaining growth, development, health, survival, and reproduction. This has prompted extensive interdisciplinary research on sleep and circadian rhythms across disciplines from physiology and medicine to human biology and physiological anthropology [16–30].

Melatonin and vitamin D biosynthesis correlate inversely with exposure to solar radiation. Vitamin D is synthesized when skin is exposed to solar UV radiation. Melatonin synthesis occurs in the pineal gland, mainly at night. Among mammals, sleep–wake cycles range widely, from just 2 h per 24 h (elephant) to 22 h per 24 h (koala), while primates range from 8 to 15 h per 24 h, and humans average 7–9 h, varying with light exposure, ecological, sociocultural, and environmental settings [16–18]. The best hypothesis for why all organisms sleep is an evolutionary trade-off between energetic investments in growth/development/self-maintenance and those in reproductive effort [18–20]. Supporting data for a self-maintenance model ranges from somatic cells conserving energy, repairing themselves, and disposing of metabolic waste to neurological cells consolidating memories and promoting dream states during sleep, to associations of poor or impaired sleep with higher risks for elevated blood pressure, cardiovascular diseases, and higher allostatic load among humans [17–24].

Among mammals, a biological clock, located in the hypothalamic suprachiasmatic nucleus, responsive to light exposure regulates species and individual circadian rhythms [25, 26]. Daily activity patterns and intra-intergroup dynamics further influence sleep–wake cycles among humans and other mammals [19, 25–27]. Among those residing in industrial settings, sleep durations over or under 7 h tend to be associated significantly with poorer health outcomes and shorter lifespans [18]. Across samples, irregular sleep–wake cycles tend to be associated with cardiovascular events [23, 24, 27, 28]. As a neuroendocrine regulator, circulating vitamin D aids in maintaining human sleep–wake cycles, influences circadian rhythms, and is active along pathways producing melatonin [29, 30].

Among mammals, sleep physiology and biorhythms vary significantly across individuals by age, physical, and social environments. For example, across human populations, whether foragers, hunters, laborers, or suburbanites, elders tend to be more active during mornings and begin their nightly sleep earlier in the evening [19, 26], while younger individuals tend to begin to sleep later and to awaken and start their daily activities later than older, a pattern suggesting the hypothesis that early rising elders have provided a group survival advantage by functioning as early morning sentinels, while late-sleeping wakeful at night youth provide a similar advantage during evenings [18, 19, 26, 27]. Such sleep–wake patterns may differ within settings wherein artificial light at night (LAN) is a prominent component of daily life. Over recent decades, use of LAN has increased geometrically, producing broad-scale light pollution, and thereby environmental settings not previously experienced by humankind, along with altering intra-intergroup socioecological dynamics (see [2, 19, 31]). Higuchi [31] suggests that high exposures to LAN may alter circadian rhythm by shifting internal circadian clocks to a nocturnal type. Light pollution likely alters circadian rhythm by reducing melatonin secretion [31, 32]. Patel [32] suggests that although light pollution at night is negatively associated with sleep outcomes within a United States sample, its effects were negligible and mainly idiosyncratic. Such observations suggest human responses to LAN and light pollution will continue as a research interest across physiological anthropology and human biology.

#### Cold

Physiological anthropologists and human biologists also share research interests in human physiological responses to environmental stressors in settings with extremes of cold, heat, and low oxygen availability. Across hot environments, endothermic mammals must eliminate excess body heat, while in cold ones, they must generate sufficient heat to maintain physiological function [33, 34]. Mammals residing in extremely cold environments have evolved multiple physiological responses to cold stress, including layered fur, hibernation, caves, and subterranean shelters, along with larger adipose tissue deposits: brown (for generating heat by non-shivering thermogenesis: NST) and white (for greater insulation). Humans also rely on sociocultural and behavioral responses unavailable to other mammals residing in cold environments, e.g., constructed shelters, warm clothing, fire, and indoor heating. Across mammals including humans, subpopulations of species residing in extreme cold environmental settings also share an adaptive response of maintaining larger and metabolically more active brown adipose tissue (BAT) deposits than members of the same and related species residing in warmer environments [33–36]. As mammals, all humans carry some amount of highly vascularized BAT containing larger numbers of mitochondria along with more connective tissue than white adipose tissue (WAT) does. During whole body cooling, BAT burns internal fat for warmth via a process of nonshivering thermogenesis (NST) [33–37]. In more northerly settings, shorter day lengths upregulate pineal gland activity, thereby increasing melatonin production, and inducing higher BAT activity [34]. Increased production of BAT may have been an adaptive response supporting mammals’ successful colonization of extreme cold settings as they migrated into northern climes [33].

Given its importance in human physiological response to cold stress in extreme environments and laboratory settings, the somatic distribution and physiology of BAT are active research arenas across physiological anthropology, human biology, and physiology [33–49]. For example, Ocobock and colleagues [40] assessed skin-surface temperatures of cold-adapted sub-Arctic Finish reindeer herders at BAT-positive and BAT-negative regions during whole-body cooling. Participants responded with an average metabolic rate increase of 8.7%, with BAT-positive regions exhibiting warmer surface temperatures than BAT-negative [40]. Similarly, comparing cold-adapted Siberian Yakutia to Evanston, Illinois residents, as expected, Levi et al. [41] observed that Yakutia participants shivered less and showed greater BAT thermogenesis during cooling. Elsewhere, over a 10-day cold exposure, both BAT and nonshivering thermogenesis (NST) increased significantly among adults [36]. Even among tropically adapted Samoans, upon cooling, locations with greater BAT deposits showed higher temperatures than those with less BAT [42]. One estimate is that among modern humans, cold exposure may increase BAT activity by 45%, and oxidative phosphorylation by 182% [43]. Given BAT’s thermogenic properties, genes promoting its increase are likely to continue experiencing positive selection among modern circumpolar [44, 45] and other northerly living populations.

Based on computed tomography, BAT deposits correlate inversely with body mass index (BMI) and are larger among women than men. In modern settings, greater BAT correlates with less visceral and hepatic WAT, central obesity, body fat, and lower serum glucose. Such results suggest that BAT upregulates energy expenditure by limiting visceral WAT [37], thereby influencing markers of metabolic health [46, 47]. Interestingly, when BAT was transplanted into wild-type mice, they showed enhanced exercise capacity and physical performance [47], indicating possible health benefits for other mammals, possibly including humans. Research on how BAT influences human health currently is an active and expanding interdisciplinary research area within not only human biology and physiological anthropology but across healthcare, medicine, and physiology [48, 49]. Yoneshiro et al. [50] observed that chronic cold exposure tends to increase BAT. However, responsiveness varies with some exhibiting no measurable increases in either BAT volume or NST. Yoneshiro and colleagues [50] suggest that this may reflect the Developmental Origin of Health and Disease (DOHaD) theory, which postulates stressors experienced during early life stages fetal to maturity may induce epigenetic changes to DNA, thereby altering activity of one’s DNA to improve phenotypic responses to stressors. Supporting this model, those with greater BAT activity tend to have been conceived more often in cold months than warm [50]. Further supporting this model, non-thermal factors including lifestyles, physical activities, sociocultural structures, exercise, hypoxia, and acquired cold adaptations may influence BAT activity and NST both directly and indirectly [51]. Additionally, lifestyle factors that expose individuals more often to cold settings may enhance BAT activity, increase NST, and thereby limit cold stress [51]. Postnatal exposures to cold environments may not only enhance BAT functionality, they also may enhance life-long metabolic function throughout adulthood, as BAT activity and quantity decline with age [50].

#### Aging

Worldwide human life expectancy currently averages 73 years, with 77% of those born surviving to 65 years [52, 53]. Across demographic and gerontological research, healthcare, and retirement programs, age 65 frequently is used to demarcate a late life/post-reproductive life history stage, a stage often characterized by physical, physiological, and cognitive declines, mobility-related injury and disability (frailty), accompanied by increasing physiological dysfunction (allostatic load), and chronic degenerative conditions. Commonly, physical frailty is assessed as an index composed of traits such as walking speed, strength, stamina, and reduced physical capabilities [54], while allostatic load is indexed by elevated or reduced physiological assessments of well-being, e.g., blood pressure, epinephrine, and cortisol [55, 56].

Human biology and physiological anthropology converge on the goal of determining and exploring causes and consequences of age-related alterations in somatic and physiological function. For example, why are higher circulating vitamin D levels associated with better health and longer survival, while lower vitamin D titters are associated with ill health [57]? In a prospective sample, Wang and colleagues [58] observed relative risks for cardiovascular disease were inversely associated with circulating vitamin D levels. Lack of exposure to sunlight among residents of higher latitudes limits their ability to synthesize vitamin D, while populations nearer the equator synthesize greater amounts [59]. Across more northerly settings in the USA, e.g., Minnesota, Wisconsin, and elsewhere, elevated cardiovascular disease mortality rates among older adults covary with their degree of vitamin D deficiency, a condition frequent among populations at higher latitudes worldwide. Conversely, vitamin D synthesis in response to UVB exposure is higher among people living at lower altitudes, particularly those nearer the equator. As discussed earlier, vitamin D modulates circulating phosphate and calcium levels, thereby influencing bone formation, and supports melatonin synthesis, thereby contributing to regulating sleep–wake cycles, while also influencing health, disease and mortality risks, and apparently slowing cardiovascular disease progression [29, 30, 58–60]. For example, among NHANES III participants, those in the lowest vitamin D quartile experienced 26% greater mortality than those in the highest [60]. When combined, such results suggest that higher circulating vitamin D may be a biomarker of physiological health among elders.

The enzyme telomerase supports extension and maintenance of telomere lengths in gametes, prenatal cells during growth and development, and cancerous cells. Among humans, average telomere lengths decline rapidly between birth and age five, continue to decline over the lifespan, and become relatively inactive in mature somatic cells as telomerase activity declines with increasing age [61], particularly among men. Conversely, among women, estrogen stimulates telomerase expression and may limit telomere attrition [62]. One suggestion is that high telomerase levels in early life and their rapid decline thereafter represent an evolutionary trade-off between tumor-suppression and tissue-repair thereby influencing late life senescent decline [63]. Complementarily, among rodents, catch-up growth following intrauterine growth restriction may promote telomere shortening leading to later development of degenerative conditions (see [64–66]).

Among humans, telomere length is a heritable trait, with telomere shortening occurring more rapidly during early life, and older fathers passing longer telomeres to their offspring [66]. One suggestion is that extension of human reproductive lifespans has contributed to increased telomere length among humans by limiting possible high fitness costs associated with premature aging [63]. As older fathers pass longer telomeres to their offspring, they also may provide a molecular signal as to possible reproductive benefits for older males in their current environmental setting [63, 65]. In stable environments, given elders often achieve high sociocultural status, older fathers are likely to produce more offspring. Conversely, longer telomeres may be selected against due to increased mutation probabilities and associations with chronic conditions [63, 65, 67, 68].

In addition to age, personal behaviors, diet, disease, environment, health, lifestyle, sociocultural settings, and physiological stress appear to negatively influence human telomere lengths [67, 69]. Conversely, lifestyle modifications and stress management may positively influence peripheral blood mononuclear cell telomerase activity [70–72], and developing offspring may adjust telomere lengths secondary to intrauterine energetic constraints (see [72]). Longer telomeres, lifespans, and reproductive spans likely were favored due to their positive associations with higher reproductive output. As suggested for sleep, telomere length decline with age may reflect an evolutionary trade-off between early life fertility/reproductive investment and late life survival.

### Physiological anthropology future directions

Physiological anthropology focuses on human adaptations to the environment and their variations. It is characterized by the study of human adaptation to various artificial environments created by modern society [31, 32]. For example, artificial light at night (LAN), our ancestors would not have experienced nights as bright as those of many modern settings. Today’s bright nights may represent an abnormal environment in the context of human history. Determining whether humans can adapt to LAN and the effects of LAN are important research topics in physiological anthropology [31, 32].

As Yasukouchi [2] stated, physiological anthropology and human biology are closely related fields that both share and diverge in their research goals, methods, and sampling strategies. Both disciplines explore influences of natural, artificial, built, and sociocultural environments on human health, well-being, productivity, survival, aging, and genetics. Within North America, anthropological research on modern human physiological variation is most prominent in human biology. Elsewhere, physiological anthropology is an independent academic discipline, focused on variation in human functional physiological responsiveness to experienced environments and technology. Shared interests range from human responsiveness to natural (e.g., cold, heat, hypoxia), physiological (e.g., energetics, nutrition, activity), and sociocultural stressors (e.g., workloads, human–machine interfaces, built environments), to gene-environment interactions. However, an additional focus of physiological anthropology is how manufactured environments and technology influence human physiological variation, techno-adaptability, and gene-environment interactions.

Both physiological anthropologists and human biologists explore adaptive physiological responses of people residing in changing and extreme environmental settings, experiencing global stressors, losses of traditional lifeways and diets, and those living with caloric insufficiency, scarcity, and abundance (see [4, 9–11]). Often, human biologists structure their research based on biocultural models integrating sociocultural, physiological, and environmental factors to understand phenotypic variability, individual well-being, and health within and across populations. Physiological anthropologists also explore these topics, while concentrating more on how technology and constructed environments influence human physiological and morphological variability (see [1, 6, 32]). The three research topics reviewed here illustrate the intertwined, yet often variable nature of research across human biology and physiological anthropology. They also accentuate commonalities. For example, in extreme cold, larger BAT deposits are protective for both humans and other mammals. Those failing to maintain BAT deposits show higher risks for metabolic dysregulation and increased body weight [73], while higher BAT enhances health and longevity among rodents. Similarly, vitamin D is necessary for proper sleep, health, and survival. Low exposure to sunlight at high latitudes contributes to low vitamin D titers, particularly among older citizens, contributing to their low bone density and elevated mortality during winter months [74, 75].

Damon’s *Physiological Anthropology* [1] documented human physiological variation as a major research trend within North American human biology, one directed at examining prior and continuing adaptive responses to experienced environmental stressors. Later, a new biocultural model of human variation developed in American human biology and physiological anthropology as research foci shifted toward political economy, and away from evolutionary physiological (see [34, 76]). Steegmann viewed such political-economic constructs as proximate and implicitly non-evolutionary, focusing on immediate causation and viewing human health and well-being as reflective of interacting biological, social, and environmental causes, but no single cause [34]. Steegmann suggested that genetic adaptation and non-genetic adaptability will be better understood if human biologists strive to determine the heritability of physiological traits related to human well-being, reproduction, and survival, goals observed within physiological anthropology, as Yasukouchi [2, 6] articulated more recently.

Current research across human biology often centers health and well-being as reflecting lived experiences within ecological and sociocultural settings secondary to environment, ecology, political dynamics, and privilege, factors best understood within a socioecological not an evolutionary context [34]. Conversely, research within physiological anthropology is designed more to document human physiological variation across and within populations within their evolutionary context [34]. Additional goals within physiological anthropology include assisting in product design and engineering, developing technological solutions to stressors, and documenting influences of technological stressors on improving human livability within cultural settings by accommodating the built environment to evolved human physiological variation. Differences between human biology and physiological anthropology explored here and documented elsewhere indicate that these are separate, but complementary scientific endeavors with overlapping goals along with each including disciplinary specific goals. Physiological anthropology concentrates more on the human-technology interface, documenting physiological responsiveness to our rapidly changing environment, AI, machine learning, and how technology alters human physiology through lived experiences. Human biology follows a human-centered research agenda, focusing more on comparative physiology across groups experiencing differing stressors within variable environmental, ecological, and sociocultural contexts. Although often exploring differing aspects of modern human variation, human biology and physiological anthropology share overlapping goals in documenting how human physiology and health are influenced by genes, environment, and sociocultural structures, including modern built environments and technology.

## Data Availability

No datasets were generated or analysed during the current study.
